# Pneumonia

**Published:** 1891-03

**Authors:** 


### PNEUMONIA!

### SYMPTOMS AND PREVENTIVES.

Pneumonia kills sometimes within forty-eight hours. There are well marked symptoms which warn you of an approach of this disease. If you know these, you ought to be able to ward off all danger.

There are three stages of this disease, in the first two of which it is possible to save a person.

In the first stage exists what is called congestion of the lungs. Of course pneumonia has its seat in the lungs. In this first stage the quantity of blood in the fine capillaries (small blood vessels) is greatly increased. These capillaries become distended, the walls are thus made thin, and some of the corpuscles, red and white, are forced through the walls into the air cells.

In an abcess of any part of the body the matter is called pus. This pus is composed of dead cells and tissue, also white corpuscles that exude from the blood vessels. The passing of the red and white, especially the white, corpuscles through the walls of the blood vessels is a common thing. The blood rushes along the blood vessels at a very rapid rate, much more rapid in the centre of the vessel than along the sides. This is a law of physics. If, for any reason, the wall of the blood vessel becomes thin, these cells gradually work their way through into surrounding tissue, or into the air cells of the lungs. As a result of the congestion (too much blood at one spot) there is inflammation. The usual cause is an exposure to the weather. A rugged, healthy man may be travelling, lose connections with trains, walk in a wet rain for miles, sit in a hotel with his wet clothes on, and become chilled through and through. A cold, influenza or la grippe may develop into pneumonia if not taken care of.

The symptom that ushers in an attack of pneumonia is a chill. If you do not have a decided chill there is not much need of anxiety. Other symptoms are headache, backache, pains in the limbs, coryza, some cough with expectoration. You probably will get a "stitch in the side," which may be due to pleurisy, or to inflammation in the lung itself. A great many have these symptoms who do not have pneumonia, but it is far safer to take care of the simple symptoms of influenza.

Beware, always, of a chill!

The second stage of pneumonia is a condition in which the mucous membrane lining the air cells is over-stimulated, and throwing out too much mucus. There is also a slight presence of the red and white corpuscles of the blood. The red corpuscles impart to the expectorated matter a rusty color. This matter is tenacious.

If the second stage is allowed to continue for any length of time, it is very likely to run into the third stage, which is that of suppuration.

In this third stage, the corpuscles of the blood pass through the walls of the blood vessels in great quantities ; the actual tissue of the lungs becomes dead, and separates—all together completely fill the at first remote air cells, later the larger air cells ; the lung solidifies, air is not allowed to enter the lungs, the blood remains impure, poisonous, and you die of suffocation.

It is almost impossible to save a person in this third stage. The first and second stages are serious and need the utmost care to escape the third stage.

To sum up in a few words, you have an attack of iufluenza, with its symptoms of headache, etc., then a decided chill. You may have the chill at once without any of the other symptoms.

Then there is congestion ; first stage. Next there is inflammation, cough and expectoration of rusty, tenacious sputum ; second stage. Lastly, suppuration, solidification of the lung ; third stage, death. There is present in all of these stages a high fever, 103° to 104°, pulse 120 to 160, and respirations 48 to the minute.

In regard to remedies, an excellent one is oxygen gas. A supply of this gas can be obtained from almost any chemist, and be carried away in a rubber bag without much loss of the gas.

One of the best remedies is ergot, as given in this combination :

| | |
|---|---|
| Fluid extract of ergot........ ....................... | 4 drachms. |
| Tincture of gelsennium.... ......................... | 2 drachms. |
| Wine of antimony......... ...................... | 2 drachms. |

Mix : take 30 drops every two hours, to be increased if necessary.

Ergot has great power in contracting the small blood vessels. Gelsennium is a safe remedy for the same purpose, and antimony acts both upon the blood vessels, and especially on the skin, in aiding perspiration.

For home treatment of pneumonia always treat the warning symptoms described above. If you get a chill, use at once the best means at hand to reduce the fever that is almost sure to follow.

Pneumonia is a disease which needs careful watching. The let-alone policy is almost sure to be fatal. You must go to bed. You must keep warm and have complete rest. You cannot be falsely brave, and declare that "it is nothing but a cold." Pneumonia is a disease in which you need plenty of nutritious food, sometimes warm stimulants, the latter being commenced early. Brandy is the best. For the high fever, cold sponging several times a day, antifebrin in five-grain doses every six hours, aconite in one-drop doses every hour.

Always maintain good general health. A well-cared-for system will stand a heavy drain upon itself. Always sleep in a room that has plenty of fresh air. Keep a window open a little on the coldest night, and a great deal on a warm night. Arrange it so that you do not feel a draught. Eat wholesome food. Do not abuse your stomach or kidneys, and take plenty of exercise.

